# Göran Garellick (March 1, 1948 – August 25, 2025)

**DOI:** 10.2340/17453674.2026.46886

**Published:** 2026-09-28

**Authors:** Henrik Malchau, together with colleagues, registry staff, and international associates

**Affiliations:** Department of Orthopedics, Sahlgrenska University Hospital, Mölndal, Sweden

**Figure:**
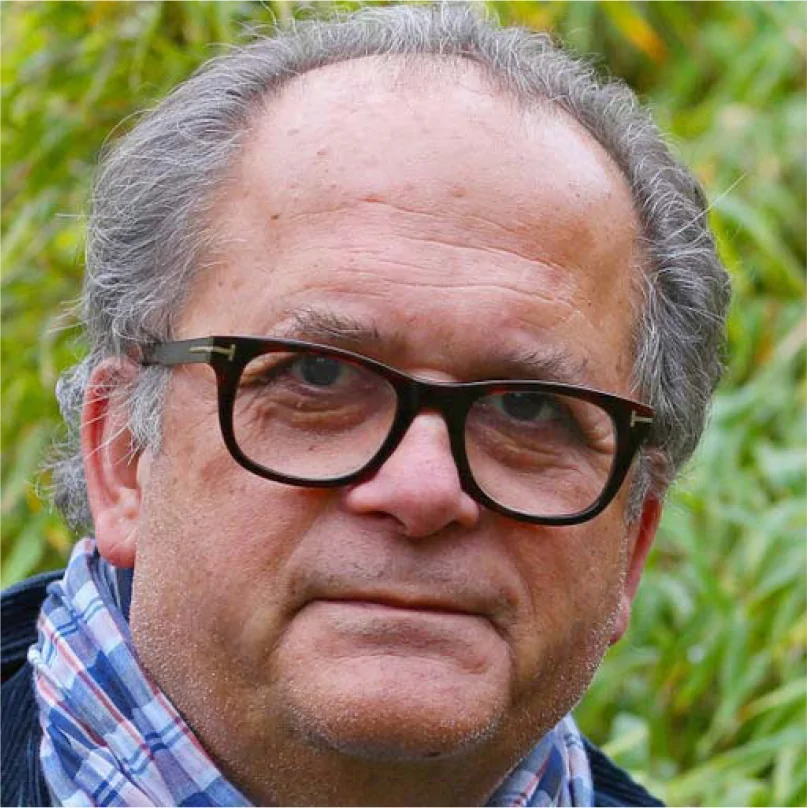
Göran Garellick (March 1, 1948 – August 25, 2025)

Göran Garellick passed away last year following a period of illness. He is survived by his wife Karin, 3 children, and 3 grandchildren.

Having worked with Göran for nearly 35 years, I wish to honor his contributions to registry-based research. At the ISAR 15th annual meeting in Lund, 2026, Göran received an honorary award—a distinction richly deserved given his efforts both in Sweden and, not least, internationally.

When we survey the global landscape of joint replacement monitoring, we see data structures that safeguard millions of patients worldwide. Yet, behind these structures stand the visionaries who built them. Göran was precisely that: an architect of modern, evidence-based orthopedic surgery. To understand Göran’s global impact, we must look to his home turf, where it all began. For many years, Göran led the Swedish Hip Arthroplasty Register (SHAR). Under his leadership, the register evolved from a local database into a world-renowned gold standard. Göran taught the orthopedic community that a registry’s purpose extends beyond the collection of passive statistics. He championed the philosophy that feedback transforms clinical practice. He consistently provided highly transparent data back to clinical units, directly driving surgical excellence, reducing revision rates, and maximizing patient safety.

Long before “Patient-Reported Outcome Measures” (PROMs) became a standard term, Göran was already applying the concept. He deeply understood that a successful hip replacement is defined by more than just a radiograph or an intact THR implant; it is defined by a patient’s ability to walk without pain and reclaim their life. Göran was instrumental in integrating the EQ-5D index and health-related quality-of-life parameters directly into the core of follow-up procedures. His work demonstrated that patient satisfaction and health economics must guide our surgical choices. This focus completely revolutionized how international orthopedic communities evaluate success.

Göran’s vision never stopped at Sweden’s borders. He was a driving force behind the establishment of the Nordic Arthroplasty Register Association (NARA), harmonizing data across Denmark, Norway, Finland, and Sweden. When ISAR was formed, Göran brought that same spirit of open collaboration, transparency, and data democratization to the global stage. He co-authored seminal accounts of registry development and mentored researchers across every continent. Moreover, he always demonstrated a unique willingness to share datasets to safeguard patient safety worldwide. Göran’s career stands as a masterpiece illustrating how data—when paired with clinical empathy and unwavering integrity—can alleviate human suffering on a massive scale. He did more than just monitor implants: he elevated the entire standard of patient care. It was with the deepest gratitude and heartfelt admiration that the International Society of Arthroplasty Registries presented Göran with a Lifetime Achievement Award.

Thank you for your leadership, your mentorship, and your enduring legacy. And Göran—thank you for the “whisky club”, all our shared travels, and the hours spent in the operating room.

May his memory be honored.


**Henrik Malchau,** together with colleagues, registry staff, and international associates *Department of Orthopedics, Sahlgrenska University Hospital, Mölndal, Sweden* *Correspondence: malchau.henrik@gmail.com*


